# Semiquantitative acid–base analysis in hypokalemic dogs with immune-mediated hemolytic anemia

**DOI:** 10.3389/fvets.2025.1563031

**Published:** 2025-05-19

**Authors:** Helen S. Philp, Steven E. Epstein, Kate Hopper

**Affiliations:** Department of Veterinary Surgical and Radiological Sciences, School of Veterinary Medicine, University of California, Davis, Davis, CA, United States

**Keywords:** autoimmune, canine, electrolyte, potassium, traditional

## Abstract

**Objective:**

To describe and compare the traditional and semiquantitative acid–base status of dogs with immune-mediated hemolytic anemia (IMHA) and hypokalemia to those with normokalemia.

**Methods:**

Medical records of dogs with IMHA from a single institution over a 10-year period from January 1st, 2012 to December 31st, 2021 were retrospectively reviewed. Dogs were included if they met diagnostic criteria for IMHA based on the 2019 ACVIM consensus guidelines and had at least 1 blood potassium concentration measurement performed within 24 h of initial presentation. The dogs were divided into normokalemic and hypokalemic groups. Hypokalemia was categorized as mild (3–3.5 mEq/L [3–3.5 mmmol/L]), moderate (2–2.9 mEq/L [2–2.9 mmol/L]), or severe (<2 mEq/L [<2 mmol/L]). Population data, clinicopathologic data, and outcome were collected and recorded. Traditional and semiquantitative acid–base diagnoses were attributed to patients with sufficient data.

**Results:**

305 client-owned dogs with IMHA met the inclusion criteria. 186 dogs (61.0%) were normokalemic and 119 (39.0%) were hypokalemic (blood potassium concentration ≤ 3.5 mEq/L [≤ 3.5 mmol/L]) on presentation. The median blood potassium concentration in the hypokalemic group was 3.2 mEq/L (3.2 mmol/L) (interquartile range: 2.8–3.4 mEq/L [2.8–3.4 mmol/L]). Hypokalemia was mild in 78/119 (65.5%) dogs, moderate in 40/119 (33.6%) and severe in 1/119 (0.84%) cases. Metabolic acidosis was the most common traditional acid–base disorder identified in both normokalemic (26/82, 31.7%) and hypokalemic (44/92, 47.8%) dogs but the proportion was significantly higher in the hypokalemic group (*p* = 0.03). The semiquantitative approach identified acid–base abnormalities in 82/83 (98.8%) hypokalemic dogs. The most common abnormalities among the hypokalemic group were an unmeasured ion effect (74/83, 89.2%) and an alkalotic albumin effect (69/83, 83.1%). In the normokalemic group, the semiquantitative approach identified acid–base abnormalities in 62/63 (98.4%) dogs with unmeasured ions (55/63, 87.3%) and an alkalotic albumin effect (42/63, 66.7%) being the most common. Survival to discharge was significantly lower in the hypokalemic dogs (85/119, 71.4%) compared to the normokalemic population (163/186, 87.6%) (*p* = 0.02).

**Conclusion:**

Hypokalemia is common in dogs with IMHA within the first 24 h of presentation and is associated with a variety of acid–base abnormalities. Hypokalemic dogs with IMHA appear more likely to develop metabolic acidosis and less likely to survive to hospital discharge.

## 1 Introduction

Immune-mediated hemolytic anemia (IMHA) results from a spontaneous immune response directed against antigens expressed on the cell surface of erythrocytes and is one of the most common autoimmune diseases of dogs (1). Mortality ranges from 7 to 50%, and IMHA is frequently associated with adverse sequelae including hypercoagulability, systemic inflammation, and organ dysfunction (2–16).

Previous studies have investigated associations between survival and various hematologic and biochemical parameters which might have prognostic value (2–7, 9, 11, 17). Hypokalemia has been documented in dogs with IMHA and was associated with increased mortality in 2 retrospective studies (7, 18), while other studies found no influence of blood potassium concentration on outcome (6, 19). An association between hypokalemia and increased mortality has already been established in a more general population of dogs presenting to an emergency room, as well as in people with acute medical conditions and the critically ill (20–22). Hypokalemia may have a direct causal relationship with mortality through detrimental effects on the cardiovascular system, nerve impulse conduction, muscle function and/or renal function (23, 24). Alternatively, hypokalemia may simply represent a nonspecific finding due to the stress of illness and associated epinephrine release (23).

The cause(s) of hypokalemia in dogs with IMHA is unknown. One case report attributed hypokalemia to distal renal tubular acidosis in 3 dogs with IMHA (25). However, the acid–base status of a larger population of dogs with IMHA has not been further investigated to the authors’ knowledge and may help to determine if renal tubular acidosis is a common causative factor of hypokalemia. Hypokalemia is frequently accompanied by acid–base disturbances due to effects on renal acid handling, aldosterone release, and the reciprocal relationship of potassium with hydrogen ions and anions such as bicarbonate and chloride (26). Semiquantitiative acid–base analysis builds on the traditional approach by consideration of some individual contributors to complex acid–base disorders (27). An improved understanding of the acid–base abnormalities in hypokalemic dogs with IMHA may offer some insight into the underlying etiology, and aid clinicians in guiding therapy for this patient population.

The aim of this retrospective study was to describe a population of dogs with IMHA and hypokalemia on presentation and to characterize their traditional and semiquantitative acid–base status and associated outcome in an effort to improve understanding of the potential underlying etiologic processes.

## 2 Materials and methods

### 2.1 Case selection

A computerized search of the medical records database at the University of California Davis, William R. Pritchard Veterinary Medical Teaching Hospital was conducted to identify dogs that were diagnosed with IMHA based on their clinical diagnosis in the medical record from January 2012 through December 2021. The case records were reviewed to verify the clinical diagnosis of IMHA. Diagnostic criteria for IMHA were based on the 2019 ACVIM consensus guidelines and required the combination of anemia, at least 2 signs of immune-mediated destruction, and at least 1 sign of hemolysis to confirm IMHA (28). Evidence of immune-mediated destruction included the combination of at least 2 of the following: spherocytes on blood smear evaluation, positive slide agglutination test and/or positive direct antiglobulin test. Alternatively, persistently positive slide agglutination after washing satisfied this criteria. Signs of hemolysis included at least one of the following: hyperbilirubinemia in the absence of an alternate hepatic or post-hepatic cause, hemoglobinemia, hemoglobinuria or erythrocyte ghosts on blood smear exam. Cases were excluded if these diagnostic criteria were not met. Repeat presentations due to persistence or recurrence of IMHA in the same patient were treated as a single case and only the first visit was included. Patients were diagnosed with primary IMHA if they had undergone a reasonable diagnostic workup to exclude an underlying trigger. Typical investigations included complete blood count, serum biochemistry, thoracic and abdominal imaging, PCR testing for tick-borne infections, echocardiography for vegetative lesions and urine culture. Cases without at least 1 blood potassium concentration measurement performed within 24 h of initial presentation were excluded. Population data including age, sex, reproductive status, bodyweight, breed, and outcome were recorded. Outcome was defined as survival to discharge or non-survival. Non-survivors included those patients that were euthanized or died in hospital. Clinicopathologic data collected included blood gas panels and serum biochemistry. The earliest measurement was utilized in cases where multiple measurements were collected.

### 2.2 Blood potassium concentration

The study included plasma potassium concentrations measured on a point-of-care analyzer^1^ and serum potassium concentrations measured as part of a serum biochemistry profile through a reference laboratory.^2^ For the purpose of this study, the term ‘blood potassium concentration’ abbreviated as [K^+^] will be used to describe all sample results for simplicity. The earliest measured [K^+^] following presentation was included for analysis. If both plasma and serum [K^+^] were measured concurrently, the point of care value was used to allow evaluation of acid–base and electrolyte abnormalities. Hypokalemia was categorized as mild (3–3.5 mEq/L [3–3.5 mmmol/L]), moderate (2–2.9 mEq/L [2–2.9 mmol/L]), or severe (<2 mEq/L [<2 mmol/L]) based on a previous study of dyskalemia in dogs and cats (22).

### 2.3 Acid–base analysis

Traditional and semiquantitative acid–base diagnoses were attributed to each patient where the relevant data was available. Traditional acid–base analysis was based on pH, PCO_2,_ standardized base excess, and anion gap and required a blood gas panel for analysis (29). The reference intervals for anion gap, venous pH, standardized base excess, and pCO_2_ from a previous publication were used (30).

Semiquantitative acid–base analysis was based on standardized base excess and the concentrations of sodium, chloride, and plasma lactate from the point of care panel and serum albumin and phosphate measured on a biochemistry panel. Serum albumin and phosphate concentrations were required to be measured on a chemistry analyzer (see text footnote 2) within 24 h of the venous blood gas measurement for this analysis with institutional reference intervals used. Calculated semiquantitative acid–base variables were derived from measured acid–base, electrolyte, and metabolite values using previously established equations (29). Bicarbonate concentration and standard base excess were calculated by the blood gas analyzer using the Henderson–Hasselbalch and van Slyke equations, respectively. The value for CO_2_ solubility in plasma used by the blood gas machine was 0.03 mmol/L/mmHg. The mid-normal values utilized in the semiquantitative formulae were determined as the central values of the appropriate reference interval for the machine utilized.

### 2.4 Statistical analysis

Statistics were performed using commercially available software.^3^ Numerical data were assessed for normality with the Shapiro–Wilk test and QQ plots. Continuous parametric data were described as mean (± standard deviation) and non-parametric data as median (25th-75th percentile). A Fisher’s exact test was performed to evaluate the relationship between categorical variables. Groups were compared with the Wilcoxon rank sum test. A *p* value < 0.05 was considered significant.

## 3 Results

### 3.1 IMHA population

A total of 305 dogs satisfied the diagnostic criteria for IMHA. The mean age of the entire population was 7.1 (±3.2) years. The predominant breeds were mixed breeds (106/305, 34.8%), chihuahuas (12/305, 3.9%), and dachshunds (12/305, 3.9%). In 2 dogs, sex was not recorded. Among the remaining 303 dogs, there were 178/303 (58.7%) females (18 intact) and 125/303 (41.3%) males (14 intact). In 119 dogs, bodyweight was not captured by the computerized search. In the remaining dogs, median bodyweight was 14.8 (6.9–26.5) kg. Of the 305 dogs with IMHA, 186 (61.0%) were normokalemic and 119 (39.0%) were hypokalemic.

### 3.2 Hypokalemic population

Among the 119 hypokalemic dogs, median [K^+^] was 3.2 mEq/L (3.2 mmol/L), (2.8–3.4 mEq/L [2.8–3.4 mmol/L]). Hypokalemia was mild in 78/119 (65.5%) dogs, moderate in 40/119 (33.6%) and severe in 1/119 (0.84%) cases. The mean age of the hypokalemic population was 6.5 (±3.1) years. The predominant breeds were mixed breeds (45/119, 37.8.%), chihuahuas (6/119, 5.0%), and poodles (6/119, 5.0%). Tere were 76/119 (63.9%) females (9 intact) and 43/119 (36.1%) males (7 intact). In 3 dogs, bodyweight was not recorded. In the remaining 116 dogs, median bodyweight was 13.3 (6.3–26.4) kg. Among the 119 dogs, 93 (78.2%) were diagnosed with primary IMHA, 5/119 (4.2%) were diagnosed with secondary IMHA and in the remaining 21/119 (17.6%) dogs definitive categorization was not possible. The most commonly reported comorbidities among the hypokalemic population included hepatopathy (18/119, 15.1%), myxomatous mitral valve disease (16/119, 13.4%), and acute kidney injury (7/119, 5.9%).

### 3.3 Outcome

There was a significant difference in outcome between groups with 85/119 (71.4%) hypokalemic dogs and 163/186 (87.6%) normokalemic dogs surviving to discharge (*p* = 0.02).

### 3.4 Total serum magnesium concentration

Total serum magnesium concentration was measured in 30/119 (25.2%) hypokalemic dogs with a mean of 2.23 (±0.42) mg/dL (0.92 [±0.17] mmol/L). Total serum magnesium concentration was measured in 21/186 (11.3%) normokalemic dogs with a median of 2.1 (1.9–2.2) mg/dL (0.86 [0.78–0.91] mmol/L). There was no significant difference between groups (*p* = 0.45).

### 3.5 Traditional acid–base analysis

Blood gas analysis was available in 174/305 (57.0%) of the total population including 92/119 (77.3%) hypokalemic dogs and 82/186 (44.1%) normokalemic dogs. Traditional acid–base values for the whole population are summarized in Table 1.

**Table 1:** Traditional acid–base analysis in 174 dogs with immune-mediated hemolytic anemia based on blood gas evaluation within 24 h of presentation and subdivided into hypokalemic and normokalemic cases.

Acid–base diagnosis	92 hypokalemic dogs *N* (%)	82 normokalemic dogs *N* (%)
Normal acid–base balance	36 (39.1)	48 (58.5)
Simple disorders		
Metabolic acidosis with normal anion gap	11 (12.0)	8 (9.8)
Metabolic alkalosis	0	1 (1.2)
Respiratory acidosis	0	2 (2.4)
Respiratory alkalosis	0	1 (1.2)
Mixed disorders		
Metabolic acidosis with normal anion gap and respiratory acidosis	3 (3.3)	5 (6.1)
Metabolic acidosis with normal anion gap and respiratory alkalosis	21 (25.6)	12 (14.6)
Metabolic acidosis with increased anion gap and respiratory alkalosis	9 (9.8)	1 (1.2)
Metabolic alkalosis and respiratory acidosis	1 (1.1)	0
Metabolic alkalosis and respiratory alkalosis	11 (12.0)	4 (4.9)

Among the 92 hypokalemic dogs with blood gas analysis available, 36/92 (39.1%) had a normal traditional acid–base status. Metabolic acidosis was the most common disorder identified, occurring in 44/92 (47.8%) of cases when both primary and mixed disorders were considered. The majority of cases with a metabolic acidosis were associated with a normal anion gap (35/44, 79.5%). Twelve of the 92 patients (13.0%) had a metabolic alkalosis.

Of the 186 normokalemic dogs with IMHA, blood gas analysis was available in 82 cases (44.1%). A normal traditional acid–base status was found in 48/82 (58.5%). When both primary and mixed disorders are considered, metabolic acidosis was the most common disorder, occurring in 26/82 (31.7%) of dogs. Most cases of metabolic acidosis were associated with a normal anion gap (25/26, 96.2%). Five dogs had a metabolic alkalosis (5/82, 6.1%) while respiratory acidosis was found in 7 cases (7/82, 8.5%) and respiratory alkalosis was identified in 18 patients (18/82, 22.0%).

There was no significant difference in the proportion of dogs with a metabolic alkalosis in the hypokalemic population when compared to those with normokalemia (*p* = 0.2) while the proportion of patients with a metabolic acidosis was higher in the hypokalemic group (47.8% vs. 31.7% [*p* = 0.03]).

### 3.6 Semiquantitative acid–base analysis

Both venous blood gas analysis and serum biochemistry results were available in 146/305 (47.9%) of the total population including 83/119 (69.7%) hypokalemic dogs and 63/186 (33.9%) normokalemic dogs. Acid–base, electrolyte and plasma lactate values for both groups are summarized in Table 2 and specific acid–base disorders are listed in Table 3.

**Table 2:** Acid–base, electrolyte, and plasma lactate values in 146 dogs with immune-mediated hemolytic anemia measured within 24 h of presentation and subdivided into hypokalemic and normokalemic cases.

Group	Hypokalemic (83 dogs)		Normokalemic (63 dogs)	
Parameter (Reference range)	Median (25th-75th percentile) in conventional units	Median (25th-75th percentile) in SI units	Median (25th-75th percentile) in conventional units	Median (25th-75th percentile) in SI units
Sodium (143–151 mEq/L; 143–151 mmol/L)	149 (146–151.5) mEq/L	149 (146–151.5) mmol/L	148 (146–150.5) mEq/L	148 (146–150.5) mmol/L
Potassium (3.6–4.8 mEq/L; 3.6–4.8 mmol/L)	3.2 (2.85–3.35) mEq/L	3.2 (2.85–3.35) mmol/L	3.9 (3.7–4.2) mEq/L	3.9 (3.7–4.2) mmol/L
Chloride (108–116 mEq/L; 108–116 mmol/L)	120 (114.5–123.5) mEq/L	120 (114.5–123.5) mmol/L	117 (114–121) mEq/L	117 (114–121) mmol/L
Corrected chloride (108–116 mEq/L; 108–116 mmol/L)	116.8 (113.9–119.1) mEq/L	116.8 (113.9–119.1) mmol/L	116.2 (113.4–117.2) mEq/L	116.2 (113.4–117.2) mmol/L
Albumin (3.4–4.3 g/dL; 34–43 g/L)	3 (2.7–3.25) g/dL	30 (27–32.5) g/L	3.1 (2.85–3.45) g/dL	31 (28.5–34.5) g/L
Phosphorus (2.6–5.2 mg/dL; 0.84–1.68 mmol/L)	3.9 (3.05–4.5) mg/dL	1.26 (0.98–1.45) mmol/L	4.2 (3.45–5.1) mg/dL	1.35 (1.11–1.65) mmol/L
pH (7.361–7.444)	7.382 (7.343–7.412)	7.382 (7.343–7.412)	7.375 (7.342–7.406)	7.375 (7.342–7.406)
PvCO_2_ (27.1–38.7 mmHg)	30.1 (26.5–34.75) mmHg	30.1 (26.5–34.75) mmHg	34 (30.2–38.05) mmHg	34 (30.2–38.05) mmHg
Bicarbonate (17.2–23.0 mEq/L; 17.2–23.0 mmol/L)	17.7 (14.8–19.65) mEq/L	17.7 (14.8–19.65) mmol/L	18.6 (16.65–20.65) mEq/L	18.6 (16.65–20.65) mmol/L
Standardized base excess (−5.5 – −0.9 mEq/L; −5.5 – −0.9 mmol/L)	−6.4 (−9.7–−4.65) mEq/L	−6.4 (−9.7–−4.65) mmol/L	−5.6 (−7.75–−3.45) mmol/L	−5.6 (−7.75–−3.45) mmol/L
Lactate (<22.5 mg/dL; < 2.5 mmol/L)	20.7 (15.3–31.1) mg/dL	2.3 (1.7–3.45) mmol/L	18.9 (15.76–30.18) mg/dL	2.1 (1.75–3.35) mmol/L
Anion gap (12–21 mEq/L; 12–21 mmol/L)	15.5 (13.2–17.75) mEq/L	15.5 (13.2–17.75) mmol/L	16.5 (14–18.9) mEq/L	16.5 (14–18.9) mmol/L
Free water effect	N/A	0.75 (0–1.38) mmol/L	N/A	0.5 (0–1.125) mmol/L
Chloride effect	N/A	−5.8 (−8.1–−2.9) mmol/L	N/A	−5.2 (−6.2–−2.4) mmol/L
Albumin effect	N/A	3.33 (2.41–4.44) mmol/L	N/A	2.96 (1.665–3.885) mmol/L
Phosphorus effect	N/A	0 (−0.35–0.49) mmol/L	N/A	−0.174 (−0.696–0.261) mmol/L
Lactate effect	N/A	−2.3 (−3.5–−1.7) mmol/L	N/A	−2.1 (−3.35–−1.75)
Sum of effects	N/A	−3.80 (−7.51 to −1.58) mmol/L	N/A	−3.77 (−6.25–−1.42) mmol/L
Unmeasured ions	N/A	−2.31 (−4.90–−0.68) mmol/L	N/A	−1.78 (−3.58–−0.136) mmol/L
Unmeasured anions	N/A	−3.8 (−5.3–−2.0) mmol/L	N/A	−1.8 (−4.3–−1.7) mmol/L
Unmeasured cations	N/A	1.3 (0.95–2.9) mmol/L	N/A	2.0 (0.93–3.3) mmol/L

**Table 3:** Semiquantitative acid–base analysis in 146 dogs with immune-mediated hemolytic anemia based on venous blood gas and serum biochemical evaluation within 24 h of presentation and subdivided into hypokalemic and normokalemic cases.

Metabolic acid–base diagnosis	83 Hypokalemic dogs *N* (%)	63 Normokalemic dogs *N* (%)
One or more acidotic processes	79 (95.2)	60 (95.2)
One or more alkalotic processes	76 (91.6)	53 (84.1)
Both alkalotic and acidotic processes	73 (88.0)	51 (81.0)
Dilutional acidosis	1 (1.2)	1 (1.6)
Contraction alkalosis	29 (34.9)	16 (25.4)
Acidotic chloride effect	51 (61.4)	32 (50.8)
Alkalotic chloride effect	1 (1.2)	1 (1.6)
Acidotic albumin effect	0 (0)	0 (0)
Alkalotic albumin effect	69 (83.1)	42 (66.7)
Acidotic phosphate effect	7 (8.4)	7 (11.1)
Acidosis with hyperlactatemia	47 (56.6)	35 (55.6)
Unmeasured ions	74 (89.2)	55 (87.3)
Unmeasured anions	62 (74.7)	43 (68.3)
Unmeasured cations	12 (14.5)	12 (19.0)

The semiquantitative approach identified acid–base abnormalities in 82/83 (98.8%) hypokalemic dogs. One or more acidotic processes were identified in 79/83 (95.2%) dogs while 76/83 (91.6%) dogs had one or more alkalotic processes. The most common abnormalities among the hypokalemic group were an unmeasured ion effect (74/83, 89.2%), alkalotic albumin effect (69/83, 83.1%), acidotic chloride effect (51/83, 61.4%), and acidosis with hyperlactatemia (47/83, 56.6%). Unmeasured anions (62/83, 74.7%) were more frequent than unmeasured cations (12/83, 14.5%).

The semiquantitative approach identified acid–base abnormalities in 62/63 (98.4%) normokalemic dogs. One or more acidotic processes were identified in 60/63 (95.2%) dogs while 53/63 (84.1%) dogs had one or more alkalotic processes. The most common abnormalities were unmeasured ions (55/63, 87.3%), an alkalotic albumin effect (42/63, 66.7%), acidosis with hyperlactatemia (35/63, 55.6%), and an acidotic chloride effect (32/63, 50.8%). Unmeasured anions (43/63, 68.3%) were more frequent than unmeasured cations (12/63, 19.0%).

## 4 Discussion

Hypokalemia was relatively common within the first 24 h of presentation in this population of dogs with IMHA, with a prevalence of 39.0%. This is similar to a previous report of hypokalemia in dogs with IMHA which found that 42% of cases had a [K^+^] < 3.6 mEq/L (< 3.6 mmol/L) on presentation (18). In most of the dogs presenting with hypokalemia in our study population, the degree of hypokalemia was mild. The study population signalment as a whole was similar to that previously reported with a mean age of 6.5 years and a predominance of females (4, 7, 8, 10–12, 14–16, 31, 32).

In this study, 85/119 (71.4%) hypokalemic dogs and 163/186 (87.6%) normokalemic dogs survived to discharge. These appear broadly similar to those previously reported in dogs with IMHA which range from 44 to 93% depending on the timeline evaluated (2, 5–8, 10–17, 33). In agreement with two previous studies of dogs with IMHA (7, 18), our study found a significant difference in outcome between dogs with normokalemia and those with hypokalemia on presentation. There are a number of potential adverse effects of hypokalemia. Potassium is essential for maintaining normal cell membrane potential, nerve impulse transmission, and muscle function. Hypokalemia leads to enhanced neuromuscular excitability via increased permeability of sodium channels in the cell membrane. Possible clinical manifestations include muscle cramps, gastrointestinal ileus, rhabdomyolysis, paralysis and even respiratory failure in severe cases (23). Hypokalemia may promote diastolic cardiac dysfunction as well as increase the risk of arrhythmias by enhancing automaticity and delaying repolarization (23). Endothelial dysfunction, and increased rates of thrombosis and platelet aggregation have also been described (20, 21). Additional adverse effects of hypokalemia may relate to the vasoactive role of potassium. For example, release of potassium from skeletal muscle enhances vasodilation and perfusion during exercise. In experimental murine models, hypokalemia has been associated with alterations in vasoactive mediators which favor renal vasoconstriction and could theoretically lead to tubular damage via intrarenal ischemia (34–36). Over time, hypokalemia can lead to both structural and functional changes in the kidneys resulting in reduced urine concentrating ability, increased ammoniagenesis, altered sodium reabsorption, and enhanced bicarbonate reabsorption. Tubulointerstitial lesions have been reported in people and cats with chronic hypokalemia (24, 37). Despite the potential negative consequences of hypokalemia, it is also possible that hypokalemia in dogs with IMHA simply represents a marker of disease severity rather than playing any direct pathophysiologic role (24).

Possible causes of hypokalemia in dogs with IMHA include stress of illness with associated cortisol and/or epinephrine release, inappetence, intravenous fluid therapy, gastrointestinal loss through vomiting and/or diarrhea, renal losses secondary to intrinsic kidney disease, medications including corticosteroids, endocrine disorders and hypomagnesemia (23, 24, 38–44). Total serum magnesium concentration did not differ between groups, making hypomagnesemia a less likely contributor to the development of hypokalemia, but given the small population in which serum magnesium concentration was measured in this study a definitive conclusion cannot be made. Ideally, ionized magnesium would have been measured. Unfortunately, the retrospective nature of this study limits our ability to determine the etiology of hypokalemia and the precise mechanisms behind hypokalemia in these dogs remain speculative. Future prospective studies with measurement of plasma cortisol, aldosterone, and evaluation of urinary potassium excretion would improve understanding of hypokalemia in dogs with IMHA. The potential for treatment to modify any association between [K^+^] and mortality over time and thus present an opportunity for intervention is also worthy of further exploration. Acid–base changes can influence [K^+^]. For example, alkalemia promotes intracellular translocation and hypokalemia (45). However, the proportion of metabolic alkalosis did not differ between the normokalemic and hypokalemic groups and as such is unlikely a significant contributor to hypokalemia in the dogs in this study. Normal anion gap metabolic acidosis was the most common acid–base abnormality and was more frequent in the hypokalemic group. Normal anion gap metabolic acidosis is common in small animal patients and may have been caused by bicarbonate loss via the kidney or gastrointestinal tract (29). One possible cause of a normal anion gap metabolic acidosis is distal renal tubular acidosis which has been documented in 3 dogs with IMHA, all of which were hypokalemic (25). Distal renal tubular acidosis results from inability of the renal tubules to excrete hydrogen ions and has been linked with both autoimmune disease and hemolytic anemia in people (23, 46–50). Interestingly, potassium depletion has also been observed to cause a metabolic acidosis in canine experimental studies which is reversible on potassium repletion (24, 51, 52). The cause appears to be a distal renal tubular acidification defect and may involve decreased aldosterone release (24, 52). Unfortunately, given the retrospective nature of this study it was not possible to further evaluate these potential mechanisms.

Semiquantitative acid–base analysis identified the presence of multifactorial metabolic acid–base abnormalities in both the normokalemic and hypokalemic populations. The most common acid–base influences identified in the hypokalemic group were an unmeasured ion effect, an alkalotic albumin effect and acidotic processes associated with hyperchloremia and hyperlactatemia. Unmeasured anions were more frequent than unmeasured cations. Although the semiquantitative approach identified unmeasured anions frequently in this population, the quantity present was relatively small. The value for unmeasured ions as determined by the semi-quantitative approach is the result of multiple calculations, making it prone to compounded error and the clinical relevance of this parameter has not been validated. Possible causes of unmeasured anions such as propylene glycol or d-lactate could not be evaluated in this retrospective study (53). Hypoalbuminemia has been reported previously in dogs with IMHA and attributed to an acute phase response, impaired hepatic function, and/or hemorrhage (9). Hypoalbuminemia may obscure the presence of unmeasured acids and an increased anion gap on traditional acid–base analysis. Hyperchloremia may reflect bicarbonate losing processes such as renal tubular acidosis or gastrointestinal losses. Hyperlactatemia is well described in dogs with IMHA and was documented in 84% of patients on admission in one study (6).

Understanding these individual processes may allow a more tailored treatment plan. For example, due to the frequent occurrence of unmeasured acids and hyperchloremia, fluid therapy using a balanced isotonic crystalloid solution may be more suitable than 0.9% saline for hypokalemic dogs with IMHA. This approach may help to prevent the acidifying and renal vasoactive effects associated with excessive chloride, especially when hypokalemia is managed with intravenous potassium chloride supplementation (54).

This study has a number of limitations, many of which are inherent to its retrospective nature. Our overall number of cases is likely an underestimation of the true prevalence of hypokalemia in dogs with IMHA. We only included cases where a diagnosis of IMHA was confirmed or supported, excluding many cases where the history, clinical findings and histopathology were very suggestive of IMHA. Measurement of [K^+^] and blood gas analysis within 24 h of presentation to the hospital were only performed within a subset of patients at the discretion of the primary clinician. It is possible that clinicians are more likely to measure electrolytes and perform blood gas analysis in animals considered to have more severe disease leading to selection bias and overestimation of theassociation between hypokalemia and mortality. In addition, serum chemistry and blood gas analysis were performed in a greater proportion of hypokalemic dogs than normokalemic dogs which may have further affected our findings. Future research in a randomly selected population with standardized protocols for measurement of [K^+^], serum chemistry, and acid–base analysis is necessary to confirm the relationship between hypokalemia and patient outcomes.

In order to better understand the potential causes of hypokalemia in this population, it would also have been ideal to measure urine pH and electrolytes at the time of blood gas analysis in all patients. Finally, blood gas and serum chemistry panels were not collected at the same time in each patient and some patients may have received treatment prior to blood sampling. Our aim was to limit the effect of this variation by only including dogs with sampling performed within the first 24 h of presentation.

## 5 Conclusion

In conclusion, hypokalemia is common in dogs presenting with IMHA and is associated with a variety of acid–base abnormalities for which the etiology is likely multifactorial. These results suggest that hypokalemic dogs with IMHA may be more likely to develop metabolic acidosis and may be less likely to survive to hospital discharge although a direct causative effect cannot be confirmed. Future prospective studies are needed to further evaluate the possible underlying causes and to determine if this effect is modified with treatment.

## Data Availability

The raw data supporting the conclusions of this article may be made available by the authors on request. Requests to access the datasets should be directed to HP, hsphilp@ucdavis.edu.

## Footnotes

^1^ABL 815, Radiometer Medical A/S, Copenhagen, Denmark.

^2^Chemistry analyzer, Hitachi 917, Roche Diagnostics, Indianapolis, IN.

^3^Graph Pad Prism v 8.0, La Jolla, CA.
